# Prevalence and Correlates of Poor Oral Hygiene among School-Going Students in Mongolia

**DOI:** 10.3390/dj9020012

**Published:** 2021-01-20

**Authors:** Javzan Badarch, Suvd Batbaatar, Edit Paulik

**Affiliations:** 1 Department of Public Health, Faculty of Medicine, University of Szeged, 6720 Szeged, Hungary; paulik.edit@med.u-szeged.hu; 2 Department of Environmental Health, National Center for Public Health, Ulaanbaatar 210349, Mongolia; suvd552001@gmail.com

**Keywords:** oral hygiene, prevalence, health survey, risk factors, Mongolia, school going students

## Abstract

Brushing at least twice a day is one of the most effective methods for the prevention of dental caries and oral diseases. The aim of the present study was to determine the prevalence and correlates of poor oral hygiene in Mongolian school-going students. A secondary analysis of nationally representative data from the 2013 Mongolian Global School-based Health Survey (GSHS) was performed. In the survey, a questionnaire was completed by 5393 students aged 12–16 years old. The prevalence of poor oral hygiene and its association with some independent variables were analyzed by frequency distribution, chi-squared test, and logistic regression. The overall prevalence of poor oral hygiene was 33%. In the multivariate analysis, male students, inadequate fruit and vegetable intake, parents’ smoking, being exposed to second-hand smoke, poor parental supervision and connectedness, physical inactivity, and sedentary behavior were significantly associated with poor oral hygiene. Meanwhile, students who ate fast food and drank carbonated soft drink were found to be less likely to be poor tooth-brushers in 2013. Various determinants were identified in connection with poor oral hygiene. Based on these findings, it is recommended that an oral health promotion program should be combined with general health promotion and lifestyle intervention programs for this target population.

## 1. Introduction

Oral health is an essential component of well-being during the whole lifetime [1]. Good oral hygiene (brushing tooth twice a day) is one of the most effective methods for the prevention of dental caries and other oral diseases [2]. The World Dental Federation and World Health Organization (WHO) have indicated that more than 200 diseases can be the consequence of dental caries [3].

In Mongolia, the first National Survey of Oral Health Status of children aged 5, 12, 15, and 18 years and adults aged 35–44 and 65–74 years in Mongolia (2013) and the Dental Survey in Mongolia (2014) showed a dramatic increase of caries among children as well as complications in adults in both urban and rural areas of the country compared to the previous study, which was conducted by the School of Dentistry, Mongolian National University of Medical Sciences in 2008 [4]. The prevalence of caries in Mongolian children is still high and has not significantly changed since 1993 [5]. According to the survey performed by Tungalag et al., 90% of the population suffers from dental diseases nationwide, and dental caries in children is the highest among all age groups [6].

The Mongolian Government approved the “National Oral Health” program in 2006. Based on the recommendation of the WHO, implementing the program was expected to reduce caries prevalence by up to 78.0% to 80.1% among 5 to 6 year olds, 60.0% to 62.0% among 12 year olds, and 70% to 71.6% in the adult population, [7].

Recommended tooth-brushing prevalence among school children was found to be 22.45% in four South-East Asian countries [8]. Poor oral hygiene among adolescents has been associated with being male [9], older age [10], sweets intake (including soft drink) [11], infrequent fruits and/or vegetables consumption [10], smoking behaviors [12], lack of protective factors including poor parental supervision [13], and unhealthy lifestyles such as inadequate exercise and sedentary leisure time [14].

Until now, no studies have investigated poor oral hygiene among Mongolian adolescents. Therefore, we aimed to investigate the prevalence and correlates of poor oral hygiene in Mongolian school-going students.

## 2. Materials and Methods

### 2.1. Participants and Procedures

In 2013, the Mongolian Ministry of Health and the Public Health Institute conducted the second nationwide Global School Based Health Survey (GSHS) in Mongolia. We carried out a secondary analysis using existing data obtained from the Mongolian GSHS 2013. The GSHS uses a standardized sampling strategy in all participating countries worldwide. The Mongolian GSHS surveyed students aged 12–16 years old (attending school grades 7–12) in nine districts of Ulaanbaatar and 21 provinces. The first-stage sampling frame involved the selection of schools. The second-stage sampling frame involved the selection of classes within the selected schools. Classes were selected randomly from all eligible classes (grades 7–12). All 59 selected schools as well as all 202 selected classes participated in the survey. All students in each selected class were given a consent form (to be signed by the student) and asked to participate voluntarily in the survey. The survey questionnaire was answered by 5393 students in grades 7–12. The school response rate was 98%, the student response rate was 89%, and the overall response rate was 88% [15].

### 2.2. Measures

The dependent variable of this analysis was poor oral hygiene. It was measured on the basis of the following question: “During the past 30 days, how many times per day did you usually clean or brush your teeth?” Response options were 1 = Did not brush my teeth, 2 = Less than 1 time per day, 3 = 1 time per day, 4 = 2 times per day, 5 = 3 times per day, and 6 = 4 or more times per day; poor oral hygiene was defined as brushing teeth less than two times per day (response codes 1 to 3) and good oral hygiene when brushing 2 or more times per day (response codes 4 to 6).

The independent variables were demographic factors, dietary behaviors, smoking habits and exposure, protective factors, physical activity, and sedentary behavior-related factors. All variables were recoded to dichotomous variables except age, which was analyzed as a continuous variable.

#### 2.2.1. Demographic Factors

Sex: “What is your sex?” (response option was 1 = male and 2 = female; recoded 0 = male (1) and 1 = female (2)).

Age: “How old are you?” (response options were 1 = 11 years old or younger, 2 = 12 years old, 3 = 13 years old, 4 = 14 years old, 5 = 15 years old, 6 = 16 years old, 7 = 17 years old and 8 = 18 years old; recoded 0 = 12 years old or younger (1–2), 1 = 13 years old (3), 2 = 13 years old, 3 = 14 years old (4), 4 = 15 years old (5) and 5 = 16 years old or older (6–8)).

#### 2.2.2. Dietary Behaviors

Carbonated soft drink intake: “During the past 30 days, how many times per day did you usually drink carbonated soft drinks, such as Coca, Pepsi Cola?” (response options were 1 = Did not drink soft drinks, 2 = Less than one time per day, 3 = 1 time per day, 4 = 2 times per day, 5 = 3 times per day, 6 = 4 times per day and 7 = 5 or more times per day; recoded 0 = yes (2–7) and 1 = no (1)).

Fast food intake: “During the past 7 days, on how many days did you eat food from a fast food restaurant, such as pizza or burger places?” (response options were 1 = 0 days, 2 = 1 day, 3 = 2 days, 4 = 3 days, 5 = 4 days, 6 = 5 days 7 = 6 days and 8 = 7 days; recoded 0 = yes (2–8) and 1 = no (1)).

Fruit intake: “During the past 30 days, how many times per day did you usually eat fruit, such as apples, grapefruit, bananas, or kiwi?” (response options were 1 = I did not eat fruit during the past 30 days, 2 = Less than one time a day, 3 = 1 time per day, 4 = 2 times per day, 5 = 3 times per day, 6 = 4 times per day and 7 = 5 or more times per day; recoded 0 = inadequate (1–4) and 1 = adequate (5–7)).

Vegetable intake: “During the past 30 days, how many times per day did you usually eat vegetables, such as carrots, cabbage, or green vegetables?” (response options were 1 = I did not eat vegetables during the past 30 days, 2 = Less than one time a day, 3 = 1 time per day, 4 = 2 times per day, 5 = 3 times per day, 6 = 4 times per day and 7 = 5 or more times per day; recoded 0 = inadequate (1–4) and 1 = adequate (5–7)).

#### 2.2.3. Smoking Behaviors

Cigarette smoking: “During the past 30 days, on how many days did you smoke a cigarette?” (response options were 1 = 0 days, 2 = 1 or 2 days, 3 = 3 to 5 days, 4 = 6 to 9 days, 5 = 10 to 19 days, 6 = 20 to 29 days and 7 = All 30 days; recoded 0 = currently smoking (2–7) and 1 = never smoking (1)).

Parental smoking: “Which of your parents or guardians use any form of tobacco?” (responses options were 1 = neither, 2 = my father, 3 = my mother, 4 = both and 5 = I do not know; recoded 0 = one or both (2–4) and 1 = none (1)).

Second-hand smoke: “During the past 7 days, on how many days did people smoke in your presence?” (response options were 1 = 0 days, 2 = 1 or 2 days, 3 = 3 or 4 days, 4 = 5 or 6 days and 5 = all 7 days; recoded 0 = yes (2–5) and 1 = no (1)).

#### 2.2.4. Protective Factors

Parental supervision: “During the past 30 days, how often did your parents or guardians check to see if your homework was done?” (response options were 1 = never, 2 = rarely, 3 = sometimes, 4 = most of the times and 5 = always; recoded 0 = no (1–2) and 1 = yes (3–5)).

Parental connectedness: “During the past 30 days, how often did your parents or guardians understand your problems and worries?” (response options were 1 = never, 2 = rarely, 3 = sometimes, 4 = most of the times and 5 = always; recoded 0 = no (1–2) and 1 = yes (3–5)).

Parental bonding: “During the past 30 days, how often did your parents or guardians really know what you were doing in your free time?” (response options were 1 = never, 2 = rarely, 3 = sometimes, 4 = most of the times and 5 = always; recoded 0 = no (1–2) and 1 = yes (3–5)).

#### 2.2.5. Physical Activity

Leisure time physical activity was assessed by asking participants: “During the past 7 days, on how many days were you physically active for a total of at least 60 min per day?” (response options were 1 = 0 days, 2 = 1 day, 3 = 2 days, 4 = 3 days, 5 = 4 days, 6 = 5 days, 7 = 6 days and 8 = 7 days; recoded 0 = physically inactive (1) and 1 = physically active (2–8)).

#### 2.2.6. Leisure Time Sedentary Behavior

This was assessed by asking participants about the time they spend mostly sitting when not in school or doing homework: “How much time do you spend during a typical or usual day sitting and watching television, playing computer games, talking with friends, or doing other sitting activities?” (response options were 1 = Less than 1 h per day, 2 = 1 to 2 h per day, 3 = 3 to 4 h per day, 4 = 5 to 6 h per day, 5 = 7 to 8 h per day and 6 = more than 8 h per day; recoded 0 = yes (3–6) and 1 = no (1–2)).

### 2.3. Data Analysis

Data analysis was performed by using IBM SPSS version 24 software. Frequency distributions were used to describe demographic characteristics of the sample. Univariable and multivariable logistic regression analyses were applied to reveal the associations between poor oral hygiene and selected independent variables.

## 3. Results

### 3.1. Description of the Study Sample

The Mongolian GSHS 2013 was conducted with a total sample size of 5393 students. One-third of the students (33%) reported to have had poor oral hygiene in the 30 days preceding the survey. The main characteristics of the sample are shown in Table 1.

### 3.2. Factors Associated with Poor Oral Hygiene

According to the univariate analysis, students who reported poor oral hygiene tended to be males and be in age group older than 12 years. They consumed carbonated soft drinks and fast food, had inadequate fruit and vegetable intake, smoked cigarettes, one or both parents were smokers, were exposed to second-hand smoke at home, and suffered from poor parental supervision and disconnectedness. Parents of these students typically did not know what their children did, and the students were physically inactive and spent more than 3 h per day sitting. All the listed factors were in a significant relationship with poor oral hygiene among Mongolian school-going students.

Multivariable analysis showed that males were 1.54 times (Adjusted Odds Ratio; (AOR) = 1.54; 95% Confidence Interval (CI) [1.36–1.75]) as likely as females to have poor oral hygiene. Concerning dietary behaviors, students who consumed carbonated soft drinks and fast food were 15% (AOR = 0.85; 95% CI [0.74–0.97]) and 26% (AOR = 0.74; 95% CI [0.65–0.84]) less likely to be associated with insufficient tooth brushing. Moreover, students who had inadequate fruit and vegetable intake were 80% (AOR = 1.80; 95% CI [1.36–2.37]) and (AOR = 1.80; 95% CI [1.59–2.21]) more likely to have poor oral health than students who ate adequate amounts of fruits and vegetables. As to smoking behaviors, students whose parents (one or both) were smokers were 1.23 times more likely to report poor tooth brushing (AOR = 1.23; 95% CI [1.08–1.40]), and those being exposed second-hand smoke were 1.22 times (AOR = 1.22; 95% CI [1.06–1.40]) more likely to report poor tooth brushing. Regarding protective factors, students whose parents did not check homework were 17% more likely to report poor dental hygiene compared to fellow students (AOR = 1.17; 95% CI [1.02–1.35]), and those whose parents did not understand trouble were 30% (AOR = 1.30; 95% CI [1.13–1.49]) more likely to report poor dental hygiene compared to fellow students. Students who were physically inactive were 1.51 times (AOR = 1.51; 95% CI [1.32–1.73]) as likely to report brushing tooth less than 2 times a day. Furthermore, students who spent sitting more than 3 h per day were 1.39 times (AOR = 1.39; 95% CI [1.22–1.58)]) as likely to have poor oral hygiene. These results are presented in Table 2.

## 4. Discussion

By assessing a national sample of school-going students in Mongolia, we found that the prevalence of poor oral hygiene was 33% (including 37% of male students and 29.5% of female students) in 2013. This result was lower than the data from the 2010 GSHS Mongolia, when almost one-third of the students (41.8% of boys and 31.6% of girls) were poor tooth brushers [16], and it was lower than the prevalence in Afghanistan adolescents (60.7%) [17]. However, the last Mongolian result was higher compared to the Cambodian adolescents (20.2%) [18].

We revealed that self-reported poor oral hygiene was in correlation with male gender, inadequate fruit and vegetable intake, one or both parents being smokers, exposure to second-hand smoke, poor parental supervision and disconnectedness, and physically inactive and sedentary behavior. In addition, fast food and carbonated soft drink consumption were protective factors for poor tooth brushing (i.e., less than 2 times a day) according to the 2013 data.

In concordance with a previous study [19], our work identified males as showing poor oral hygiene behavior (less frequent tooth brushing) more frequently. It is explained that girls were more considerate of their body and appearance, and thus for their oral health, than boys.

High consumption of soft drinks among both younger and older adolescents was described as a predictor of poorer oral health and unhealthier lifestyle compared to those with lower consumption [20]. In contrast to that, findings of the current study showed that frequent carbonated soft drink and fast food consumption was a protective factor for tooth brushing less than 2 times a day.

This study confirmed the association between inadequate fruit and vegetable intake and poor dental hygiene, indicating that among young people, the consumption of unhealthy foods (lacking fruits and vegetables) is a part of wrong oral and general health behavior. It is also possible that low parental control may result in a higher prevalence of inadequate fruit and vegetable intake and poor oral health [14].

Furthermore, in this study, smoking behaviors (parental smoking and second-hand smoke exposure) were higher among poor tooth brushers. Adolescents with good oral health behavior tend to avoid smokers, thus reducing their second-hand smoke (SHS) exposure [8]. Additionally, adolescents who have good oral and general hygiene behavior may have better knowledge of the hazardous effects of smoking and passive smoke and, therefore, they might tend to avoid SHS exposure compared to those who smoke.

Parental involvement appeared to be determinant in several health behaviors, including oral health among adolescents. Socio-economic changes also affect parental care, as modern parents need to work more, cannot supervise children, and sometimes, they do not know about their children’s general and dental hygiene problems during the adolescent development. The results presented here indicate that a low level of parental bonding is associated with poor oral hygiene in adolescents, similarly to the findings of Hamilton et al. [21].

Some previous studies have highlighted the relationship between oral and general health behaviors. Regarding general health behaviors, this study found an association between being physically inactive, sedentary leisure time, and poor tooth brushing [17]. Unhealthy lifestyles may lead to worse poor oral and general hygiene.

The Government of Mongolia ratified “Healthy Teeth-Healthy Child” national program in 2018 to address dental diseases. This program will be implemented until 2023: including dental checkup, giving advice, caries treatment, root canal treatment, fluoride varnish, and tooth extraction to children ages 2 to 12. A total of 121,000 children had been provided oral health services, examinations, and advice by June 2020 [22].

Many countries have introduced effective school-based programs to improve the oral health of children. School policies and practices on healthy diet, particularly policies on sugar intake, that ensure healthy foods and drinks are provided in all areas (urban and rural), serving to promote healthy dietary behaviors from an early age [23].

## 5. Strengths and Limitations

GSHS was a large, nationally representative survey, the data of which were collected by the WHO and the Centers for Disease Control and Prevention (CDC). The GSHS is globally recognized and implemented providing highly generalizable data and findings. Schools and students were randomly selected from both urban and rural areas. Nonetheless, the findings in our study should be interpreted as having several limitations. First, there are no reliability and validity studies examining GSHS items within the context of Mongolian culture. Second, the cross-sectional nature of the study means that at one point in time, we could not assess the characteristics (e.g., oral hygiene) of students who were absent that day. Third, data were collected based on self-report, which may have biased the results.

## 6. Conclusions

Using a large and representative sample of school-going students in Mongolia, this study found the prevalence of poor oral hygiene among school-going students in Mongolia to be 33%. Factors such as male gender, inadequate fruit and vegetable intake, parents smoking, exposed second hand-smoke, poor parental supervision and disconnectedness, being physically inactive, and sitting more than 3 h a day are risk factors; meanwhile, carbonated soft drink and fast food intake are protective factors of poor oral hygiene among school-going students in Mongolia. Based on these findings, it is recommended that oral health promotion programs should be combined with general health promotion lifestyle intervention programs for this target population.

## Figures and Tables

**Table 1:** Sample characteristics associated with poor oral hygiene among school going students in Mongolia (univariable logistic regression).

	Poor Oral Hygiene		UAOR	95% CI	*p* Value
Variables	N	%			
**Demographic factors**					
Gender					
Male (n = 2516)	928	37	1.40	1.25–1.57	<0.001
Female (n = 2854)	839	29.5	1.00		
Age* (increase 1 year of age)			1.06	1.02–1.10	0.003
12 y.o or younger (n = 652)	196	30.1			
13 years old (n = 1102)	340	30.9			
14 years old (n = 994)	315	31.9			
15 years old (n = 1017)	359	35.3			
16 y.o or older (n = 1615)	559	34.7			
**Dietary behaviors**					
Carbonated soft drink					
Yes (n = 1785)	529	29.9	0.80	0.71–0.91	<0.001
No (n = 3594)	1240	34.5	1.00		
Fast food intake					
Yes (n = 2936)	876	30	0.74	0.66–0.84	<0.001
No (n = 2416)	878	36.4	1.00		
Fruit intake					
Inadequate intake (n = 4918)	1677	34.2	2.18	1.70–2.78	<0.001
Adequate intake (n = 447)	85	19.2	1.00		
Vegetable intake					
Inadequate intake (n = 4047)	1484	36.7	2.15	1.86–2.50	<0.001
Adequate intake (n = 1302)	274	21.2	1.00		
**Smoking behaviors**					
Cigarette smoking					
Currently smoking (n = 456)	187	41.5	1.49	1.22–1.81	<0.001
Never smoke (n = 4888)	1571	32.2	1.00		
Parental smoking					
One or both (n = 2397)	875	36.6	1.35	1.20–1.52	<0.001
None (n = 2889)	861	29.9	1.00		
Passive smoking					
Yes (n = 3237)	1153	35.8	1.40	1.24–1.58	<0.001
No (n = 2113)	599	28.4	1.00		
**Protective factors**					
Parental supervision					
No (n = 1710)	661	38.8	1.47	1.30–1.66	<0.001
Yes (n = 3653)	1096	30.1	1.00		
Parental connectedness					
No (n = 2709)	1015	37.6	1.54	1.37–1.73	<0.001
Yes (n = 2642)	739	28.1	1.00		
Parental bonding					
No (n = 1718)	644	37.6	1.36	1.20–1.53	<0.001
Yes (n = 3599)	1102	30.7	1.00		
**Physical activity**					
Inactive (n = 1657)	650	39.3	1.51	1.34–1.70	<0.001
Active (n = 3687)	1103	30	1.00		
**Sedentary behavior**					
Yes (n = 2342)	831	35.6	1.23	1.10–1.38	<0.001
No (n = 3026)	930	30.8	1.00		

* Age: continuous variable in logistic regression analysis. UAOR: UnAdjusted Odds Ratio. 95% CI: 95% Confidence Interval. yo: years old.

**Table 2:** Sample characteristics associated with poor oral hygiene among school-going students in Mongolia (multivariable logistic regression).

Variables	AOR	95% CI	*p* Value
**Demographic factors**			
Male gender	1.54	1.36–1.75	**<0.001**
Increase one year of age *	1.00	0.96–1.05	0.813
**Dietary behaviors**			
Carbonated soft drink intake	0.85	0.74–0.97	**0.023**
Fast food intake	0.74	0.65–0.84	**<0.001**
Inadequate fruit intake	1.80	1.36–2.37	**<0.001**
Inadequate vegetable intake	1.80	1.59–2.21	**<0.001**
**Smoking behaviors**			
Currently smoking	1.20	0.96–1.51	0.106
One or both parents smoking	1.23	1.08–1.40	**0.002**
Exposed second-hand smoke	1.22	1.06–1.40	**0.005**
**Protective factors**			
Poor parental supervision	1.17	1.02–1.35	**0.023**
Parental disconnectedness	1.30	1.13–1.49	**<0.001**
Poor parental bonding	1.03	0.89–1.20	0.609
**Physically inactive**	1.51	1.32–1.73	**<0.001**
**Sedentary behavior**	1.39	1.22–1.58	**<0.001**

* Age: continuous variable; AOR: Adjusted Odds Ratio; 95% CI: 95% Confidence Interval.

## Data Availability

This study used the Mongolian GSHS 2013 dataset publicly available on the website of CDC and WHO https://www.who.int/ncds/surveillance/gshs/mongoliadataset/en/ (accessed on 24 September 2018).
